# Construction of a Large Size Human Immunoglobulin Heavy Chain Variable (VH) Domain Library, Isolation and Characterization of Novel Human Antibody VH Domains Targeting PD-L1 and CD22

**DOI:** 10.3389/fimmu.2022.869825

**Published:** 2022-04-06

**Authors:** Zehua Sun, Wei Li, John W. Mellors, Rimas Orentas, Dimiter S. Dimitrov

**Affiliations:** ^1^ Center for Antibody Therapeutics, Division of Infectious Diseases, Department of Medicine, University of Pittsburgh Medical School, Pittsburgh, PA, United States; ^2^ Abound Bio, Pittsburgh, PA, United States; ^3^ Ben Towne Center for Childhood Cancer Research, Seattle Children’s Research Institute, Seattle, WA, United States; ^4^ Department of Pediatrics, University of Washington School of Medicine, Seattle, WA, United States

**Keywords:** antibody VH domains, CD22, PD-L1, library construction, antibody domain drug conjugations (DDC)

## Abstract

Phage display is a well-established technology for *in vitro* selection of monoclonal antibodies (mAb), and more than 12 antibodies isolated from phage displayed libraries of different formats have been approved for therapy. We have constructed a large size (10^11) human antibody VH domain library based on thermo-stable, aggregation-resistant scaffolds. This diversity was obtained by grafting naturally occurring CDR2s and CDR3s from healthy donors with optimized primers into the VH library. This phage-displayed library was used for bio-panning against various antigens. So far, panels of binders have been isolated against different viral and tumor targets, including the SARS-CoV-2 RBD, HIV-1 ENV protein, mesothelin and FLT3. In the present study, we discuss domain library construction, characterize novel VH binders against human CD22 and PD-L1, and define our design process for antibody domain drug conjugation (DDC) as tumoricidal reagents. Our study provides examples for the potential applications of antibody domains derived from library screens in therapeutics and provides key information for large size human antibody domain library construction.

## Introduction

Phage display is a well-established technology, widely used for displaying antibody domains or peptides on the surface of bacteriophage and subsequently used in different methods for *in vitro* selection (1, 2). Antibody or antibody domain (Fab, ScFv and VH/VL) library construction combined with phage display facilitates high-throughput screening, and serves as the crucial stage in the retrievals of biologically relevant binders, since multiple factors such as diversity and size affect the quality of a certain library. At the onset of the SARS-CoV-2/COVID19 pandemic in 2020, we isolated a panel of potent neutralizing binders from our phage-displayed libraries, within two weeks of obtaining the spike receptor binding domain (RBD) sequence, highlighting the importance of a high quality phage-displayed antibody libraries (3–5).

Antibody domains have multiple applications, such as designing as Chimeric Antigen Receptors (CARs) or antibody drug conjugations (ADCs) (6, 7). The first FDA approved bivalent variable-domain-only immunoglobulin fragment, Caplacizumab, was used as inhibitor to block the interaction between Von Willebrand factor multimers and platelets (8). The first instance of CAR T employing a human VH domain targeting the CD33 antigen for the treatment of acute myeloid leukemia was reported in 2008 (6). Various single domain binders were reported as neutralizers against SARS-CoV-2 and other infectious diseases (9, 10). Antibody domain drug conjugates (DDC) are yet to be reported.

Cluster of differentiation-22 (CD22) is a B-cell-specific transmembrane glycoprotein, which has emerged as an attractive target for monoclonal antibody (mAb) based therapy of B cell malignancies (11). ADC therapy against CD22 is a promising therapeutic approach to B cell malignancies which conjugates mAbs with cytotoxic agents (12). Active selection agents recognizing only one antigen on leukemia cells can cause antigen-loss relapse, which is a potential limitation of potent CD22-targeted immunotherapies (13). Multispecific duoCAR-T cells that target CD19, CD20, and CD22 show a promising effect to prevent antigen loss mediated relapse or the down regulation of target antigen in patients with B cell malignancies (7). Blockade of the immune checkpoint such as programmed cell death 1 (PD-1)/programmed death-ligand 1 (PD-L1) augments anti-tumor immunity and induces durable responses in patients bearing up with solid cancers (14). But the combination of ADC therapies against PD-L1 and CD22 on clinical efficacy in B cell malignancies are sparse (15, 16).

We constructed an initial human VH domain phage-displayed library in 2008 (17, 18). Subsequently, we have been continuously optimizing the construction of human VH domain libraries at multiple levels, including primer optimization to increase the diversity, scaffold screening and characterization to reduce toxicity, as well as to lower the risk of aggregation in purified binders. In the present study, we discuss the construction of a large-size human antibody VH domain library, and characterize the quality of the library by bio-panning against two different antigens: TGFβ1 and mesothelin. Further, one PD-L1 specific VH domain and one CD22 specific VH domain were selected for domain drug conjugate (DDC) design, as an example of the application of human antibody VH domains.

## Results

### Amplification of Full-Length VH Gene Repertoires From Human Peripheral Blood Monocytes (PBMCs)

**Figure 1:**
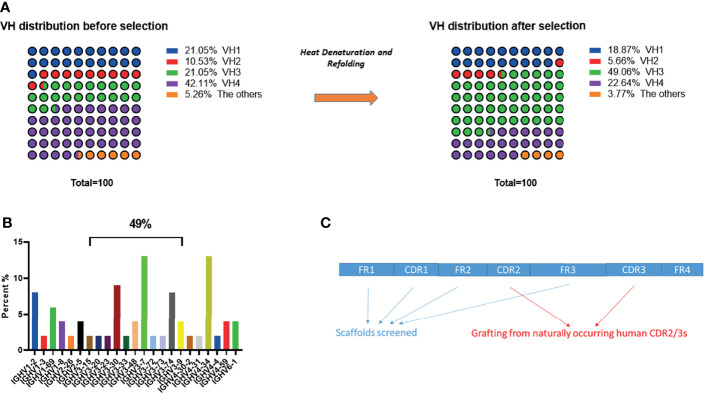
VH segments analysis and grafting of CDR2/3s. **(A)** General frequency distribution of VH gene segments before and after heat denaturation and refolding screening. **(B)** Detailed distribution of enriched VH gene segments after heat denaturation and refolding screening. **(C)** Schematic view of CDR2/3 grafting.

There are two considerations for using primary human PBMCs for the direct amplification of naturally occurring full length antibody variable heavy-chain (VH) gene repertoires and selection of scaffolds for library construction: (1) to minimize the workload of humanization; and (2) to avoid introducing immunogenic epitopes. We and others have reported that several VH sub-families such as VH1-69, VH3-7, VH3-15, VH3-23, VH4-34 and VH6-1 showed high frequency in the memory compartment of the immune repertoire obtained from healthy donors (4, 19–22). We collected human PBMCs from 10 healthy donors. Full length natural occurring VH genes were amplified by conserved region primers to construct a small size (10^8) VH domain library. Primers were designed according to the alignments from international ImMunoGeneTics information system^®^ (IMGT, http://www.imgt.org), and are displayed in **Table 1** (23, 24). We randomly sequenced 100 clones and no repeats were observed. The dominant sub-families in our VH library (**Figure 1A**) were consistent with the previous reports mentioned above.

**Table 1:** Primers for amplification of natural occurring full length VH gene repertoires.

Forward primers fornaturally occurring human VH fragments	Sequences (5’-3’)	Antibody VH sub-families amplified
VH-F1	CAG RTG CAG CTG GTG CAR TCT GG	IGHV 1
VH-F2	SAG GTC CAG CTG GTR CAG TCT GG	IGHV 1
VH-F3	CAG RTC ACC TTG AAG GAG TCT GG	IGHV 2
VH-F4	SAG GTG CAG CTG GTG GAG TCT GG	IGHV 3
VH-F5	GAG GTG CAG CTG GTG GAG WCY GG	IGHV 3
VH-F6	CAG GTG CAG CTA CAG CAG TGG GG	IGHV 4
VH-F7	CAG STG CAG CTG CAG GAG TCS GG	IGHV 4
VH-F8	GAR GTG CAG CTG GTG CAG TCT GG	IGHV 5
VH-F9	CAG GTA CAG CTG CAG CAG TCA GG	IGHV 6
**Reverse primers for** **naturally occurring human VH fragments**	**Sequences (5’-3’)**	**Antibody VH sub-families amplified**
VH-R1	CTG GTC ACY GTC TCC TCA	IGHJ1, IHGJ2, IGHJ4, IGHJ5
VH-R3	ATG GTC ACC GTC TCT TCA	IGHJ3
VH-R6	ACG GTC ACC GTC TCC TCA	IGHJ6

### Scaffolds Selection for Library Construction

We identified relatively stable VH scaffolds from a small VH phage-displayed library (10^8) by a heat denaturation and refolding strategy (4, 25). After 3 rounds of panning, output clones were enriched according to plate titration. We randomly sequenced 100 clones, and VH genes in VH3 family were largely enriched, composing up to 49% of sequenced genes (**Figure 1B**) (4, 18). We then sequenced more clones and evaluated the aggregation of the most enriched clones by size exclusion chromatography (SEC) and dynamic light scattering (DLS). The five most enriched clones (1 clone from VH 3-7, 2 clones from VH 3-30 and 2 clones from VH 4-34) were finally selected for construction of a large size VH domain library.

### Construction of a Large Size Human VH Antibody Domain Library

After selection of aggregation resistant scaffolds, we constructed a large size human VH domain library by grafting naturally occurring CDR2s and CDR3s from human PBMCs (from 50 healthy donors) (**Figure 1C**) (4, 18). Grafting of human CDR3 is an important step, since studies have verified that CDR3 of the VH domain is sufficient to distinguish between a variety of haptens and protein antigens with surprisingly high affinities (26). We optimized the primers for grafting naturally occurring human CDR3 (**Table 2**), and observed that the optimized set of primers largely increased the diversity of the library constructed. The size of the final VH phage-displayed library constructed (after grafting) was estimated to be 1.3 × 10^11^, with no repeated sequences found in 100 random sequenced clones, indicating a good diversity. 80% of the clones were productive.

**Table 2:** Primers for CDR2 and CDR3 gene grafting.

Forward primers fornaturally occurring human CD3 grafting	IMGT numbering 99 100 101 102 103 104 (5’-3’)	Antibody VH sub-families amplified
ICAT6e-F1	ACR GCY TTR TAT TAC TGT	IGHV 3–9, 3–20, 3–43
ICAT6e-F2	ACA GCC AYR TAT TAC TGT	IGHV 1–45, 2–5, 2-26, 2-70,5–*, 7–81
ICAT6e-F3	ACR GCY GTR TAT TRC TGT	IGHV 3-7, 3-11, 3-13, 3-15, 3-33, 3-66, 3-69, 4-4, 4-28, 4-30, 4-31, 4-34, 4-38, 4-39, 4-59,4-61, 6-*, 7-4
ICAT6e-F4	ACR GTC GTG TRT TAC TGT	IGHV 1-2
ICAT6e-F5	ACA GTT GTG TAC TAC TGT	IGHV 3-62
ICAT6e-F6	ACG GCC KYG TAT YAC TGT	IGHV 1-8, 1-18, 1-24, 1-46, 1-58, 1-69, 3-22, 3-38, 3-41, 3-49, 3-53, 3-71, 3-72, 3-73, 7-34
ICAT6e-F7	RTG GMC GTG TAT GGC TRT	IGHV 3-32
ICAT6e-F8	ACA GCT GTG TGT TAC TGT	IGHV 3-30-52
ICAT6e-F9	AYS GCC ATG TAT TAC TGT	IGHV 1-45, 5-10, 7-81
ICAT6e-F10 (optional)	TCG GCT GTG TAT TAC TGG	IGHV 1-68
ICAT6e-F11 (optional)	ATG ACC GTG TAT TAC TGT	IGHV 3-52
ICAT6e-F12	AYG GCT GTG TAT TAY TRT	IGHV 1-3, 1-38, 3-16, 3-19, 3-21, 3-29, 3-30, 3-35, 3-47, 3-48, 3-64, 3-74, 3-NL1
**Forward primers for** **naturally occurring human CD2 grafting**	**IMGT numbering 47 48 49 50 51 52** **(5’-3’)**	**Antibody VH sub-families amplified**
ICAT6e-F13	GGA CAA VGS CTT GAG TGG	IGHV 1–2, 1–3, 1–8, 1–18, 1–45, 1–46, 1–58, 1–69, 6–1, 7–*
ICAT6e-F14	GGA CAA VCS CTT GAG TGG	IGHV 1-45
ICAT6e-F15	GGV AAR GCC CTG GAG TGG	IGHV 2-5*, 2-26*, 2-70*
ICAT6e-F16	GGV AAR GGN CYG GAR TGG	IGHV 2-*, 3-*, 4-*, 5-*
ICAT6e-F17	TCG AGA GGC CTT GAG TGG	IGHV 6-*
**Reverse primers for** **naturally occurring human CD2 grafting**	**IMGT numbering 77 78 79 80 81 82** **(5’-3’)**	**Antibody VH sub-families amplified**
ICAT6e-R1	ACC ATB WCY ARG RAC ACV	IGHV 1-2*, 1-24*, 1-3*, 1-38*, 1-45*, 1-46*, 1-58*, 1-8*, 2-5*, 2-26*, 2-70*
ICAT6e-R2	RCC ATC TCC AGR GAY AAY	IGHV 3-11*, 3-20*, 3-21*, 3-23*, 3-30*, 3-30-3*, 3-33*, 3-38*, 3-43*, 3-47*, 3-48*, 3-64*, 3-66*, 3-69*, 3-7*, 3-74*, 3-9*, 3-NL1*
ICAT6e-R3	AYC ATC TCM AGA GAH RRT	IGHV 3-13*, 3-15*, 3-16*, 3-19*, 3-22*, 3-35*, 3-49*, 3-71*, 3-72*, 3-73*
ICAT6e-R4	ACC ATR TCM GTA GAC AVG	IGHV 4–*
ICAT6e-R5	ACC ATC TCA GCY GAC AAG	IGHV 5–*
ICAT6e-R6	ACC ATC AAC CCA GAC ACA	IGHV 6–*
ICAT6e-R7	GTC TTC TCC WTG GAC ACC	IGHV 7–*

*The rest of VH genes in the same subfamily.

### Bio-Panning of the Constructed Library Against Human Recombinant TGFβ1 and MSLN

**Figure 2:**
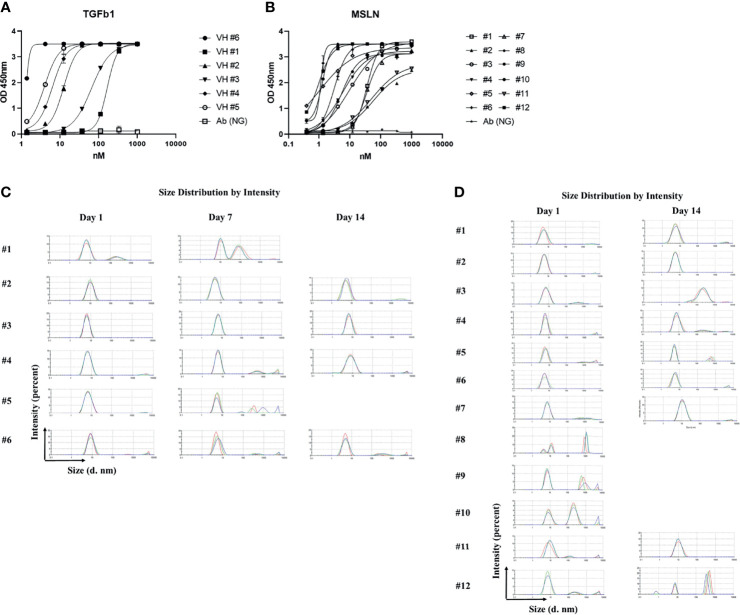
Characterization of constructed library by bio-panning against TGFb1 and MSLN. **(A)** ELISA of binders against TGFb1; **(B)** ELISA of binders against MSLN; **(C)** Dynamic light scattering analysis for evaluation of the aggregation propensity of the TGFb1 specific VH domains; 3 different colors represent 3 different repeats; **(D)** Dynamic light scattering analysis for evaluation of the aggregation propensity of the MSLN specific VH domains, 3 different colors represent 3 different repeats.

This library was panned against human recombinant Transforming Growth Factor-β1 (TGFβ1), and a panel of binders was obtained with half maximal effective concentration (EC_50_) ranging from 0.5 nM to 100 nM (**Figure 2A**). We performed 3 rounds of panning, which will be sufficient for enrichment of antigen-specific binders. The yield of both antibody VH domains ranged from 4 to 8 mg, from 100 ml 2x YT medium culture. To test aggregation, we incubated these binders at 3-5 mg/ml at 37°C for 24 hours and 7 days, and measured the aggregation by DLS (**Figure 2C**). 83% of binders showed aggregation resistance (aggregation percent < 5%) after 24 hours incubation while 67% of binders showed aggregation resistance after 7 days incubation. We kept incubating of aggregation resistant binders for another 7 days at 37°C, and all the binders showed durable aggregation resistance. We panned the library against another antigen, human recombinant mesothelin (MSLN) (by 3 rounds of panning), and created another panel of binders showing EC_50_s ranging from 1 nM to 200 nM (**Figure 2B**), and measured the aggregation of binders by DLS after incubation at 37°C for 24 hours. 75% of binders (9 out of 12) showed aggregation less than 5%. The 9 aggregation resistant binders were incubated for another 13 days at 37°C, and 5 of the candidates showed persistent aggregation resistance, with aggregation accounting for less than 5% of the binder profile (**Figure 2D**).

### Characterization of PD-L1 Specific VH Domain 1-16-3

**Figure 3:**
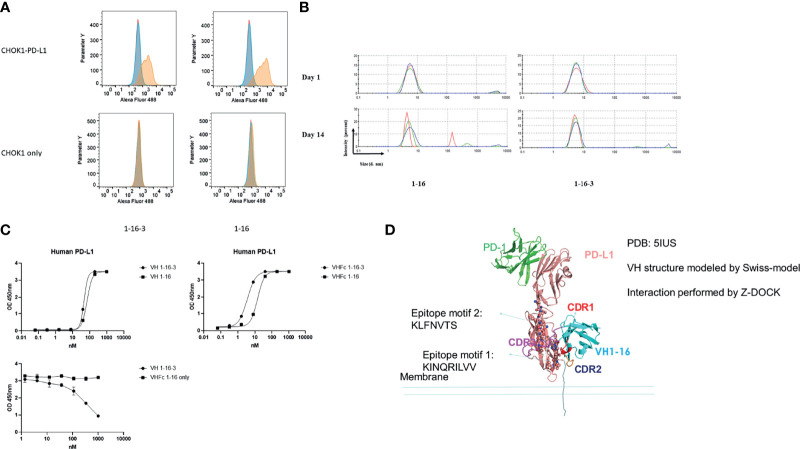
Characterization of VH domains 1-16 and 1-16-3. **(A)** Flow cytometry analysis for binding of VH 1-16 and 1-16-3 to PD-L1 expressed on the surface of CHOK1 cells; blue peaks represent cells stained with AF488 conjugated secondary antibody only, red peaks represent cells stained with isotype control and then AF488 conjugated secondary antibody, yellow peaks represent cells stained with VH domains and then AF488 conjugated secondary antibody; **(B)** Dynamic light scattering analysis for evaluation of the aggregation propensity of domain s domains 1-16 and 1-16-3; Curves with different color are different repeats; **(C)** ELISA of 1-16 and 1-16-3 against PD-L1 with or without human IgG1 Fc, and their competition; **(D)** Structural modelling of PD-L1 with VH 1-16. Epitopes were labeled in the figure.

To date, there are several anti-human PD-L1 drugs approved by FDA (27). Most of them function in blocking the PD1/PD-L1 binding interface. We quickly (only 2 rounds of panning) isolated one binder (nominated 1-16) with specific binding to PD-L1. Epitope mapping demonstrated that binders 1-16, targeted the membrane proximal domain of PD-L1, which is far away from known PD1 binding epitopes (**Figure 3D**). We further modified the CDR3 of 1-16 by grafting and mutagenesis, and acquired one variant of 1-16, nominated 1-16-3, which showed improved binding and aggregation resistance. Surface Plasmon Resonance (SPR) was further performed to measure the equilibrium dissociation constant (K_D_) of parental domain 1-16 and variant 1-16-3 to human recombinant PD-L1 (**Table 3**). After conversion to VH-Fc format to further increase the avidity, 1-16-3 showed an EC_50_ less than 10nM. PD-L1 positive CHO-K1 cells and PD-L1 negative CHO-K1 cells were used for confirming the cell specific binding for both VH 1-16 and VH 1-16-3 by flow cytometry (**Figure 3A**). The maturation of 1-16 to 1-16-3 did not block the cell specific binding nor shift the epitope since they competed to each other in a competition ELISA (**Figure 3C**). VH 1-16-3 has an improvement of aggregation resistance after incubation at 37°C for both 24 hours and 7 days by DLS (**Figure 3B**). In both VH and VH-Fc format, 1-16-3 shows an increase in binding to PD-L1 (**Figure 3C**). Both 1-16 and 1-16-3 cannot neutralize PD-L1 in a PD-1/PD-L1 Blockade Bioassay (data not shown), indicating the PD-L1 non-neutralization epitope is targeted by 1-16-3 (**Figure 3D**).

**Table 3:** SPR of PD-L1 specific VH domains 1-16 and 1-16 derived 1-16-3.

Binders	KD (M)	Ka (1/Ms)	Ka Error	Kd (1/s)	Kd Error
1-16	6.2 e-8	2.9 e4	2.2 e3	1.8 e-3	1.9 e-4
1-16-3	4.8 e-8	2.8 e4	9.4 e2	1.4 e-3	8.8 e-5

### 1-16-3 Based DDC Killed CD22 Positive Leukemia Cells Alone, or in Combination With CD22 Specific VH Domain E1-2 Based DDC

**Figure 4:**
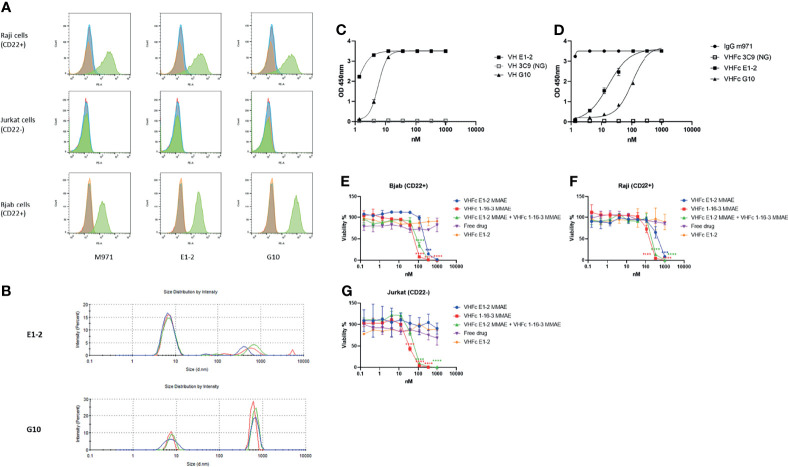
Characterization of VH domains E1-2 and DDC design an example of the potential application. **(A)** Flow cytometry analysis for binding of domains to CD22 expressed on the surface of Raji cells and Bjab cells (Jurkat cells as negative control); yellow peaks represent unstained cells, blue peaks represent cells stained with PE conjugated secondary antibody only, red peaks represent cells stained with isotype control and then PE conjugated secondary antibody, green peaks represent cells stained with VH domains or m971 (positive control), and then PE conjugated secondary antibody; **(B)** Dynamic light scattering analysis for evaluation of the aggregation propensity of domains E1-2 and G10 after 24 hours incubation at 37°C; **(C, D)** ELISA of E1-2 and G10 against CD22 with or without human IgG1 Fc; **(E–G)** 1-16-3 and E1-2 based antibody domain drug conjugates in killing CD22 positive leukemia cells. Significance was tested using one-way ANOVA, followed by the tukey’s multiple *post hoc* test. ****P < 0.0001; ***P < 0.001 versus antibody isotype control (VH-Fc E1-2) at each concentration.

CD22, which is overexpressed in B cell malignancies, is a target of great interest. VH-Fc 1-16-3 was conjugated with MMAE for a DDC study exploring killing of CD22 positive leukemia cells. For control purposes, we identified two human VH domains (E1-2 and G10) that specifically bind human recombinant CD22. Cell specific binding of both domains were measured on two different B cell lines (Raji and Bjab) and one T cell line (Jurkat) by flow cytometry. The previously identified IgG1 antibody m971 served as positive control (28, 29). Both E1-2 and G10 can bind to Raji (CD22+) and Bjab (CD22+) cells, but cannot bind to Jurkat cells (CD22-) (**Figure 4A**). We measured the aggregation of both E1-2 and G10 by DLS. G10 aggregated after incubation 37°C for 24 hours (**Figure 4B**). In contrast to G10, VH E1-2 showed less than 5% aggregation after incubation at 37°C for 24 hours by DLS (**Figure 4B**), and an EC_50_ below 10 nM by ELISA (**Figure 4C**). The equilibrium dissociation constant (KD) of VH domain E1-2 was measured by SPR (**Table 4**). After conversion to VH-Fc format, the EC_50_ of VH-Fc E1-2 was reduced, most likely due to the effect of the orientation of VH domains by human Fc protein (**Figure 4D**). We also conjugated VH-Fc E1-2 to MMAE for DDC study. VH-Fc E1-2 showed moderate killing of CD22 positive tumor cells, while VH-Fc 1-16-3 showed approximately 10-fold greater potency in comparison to VH-Fc E1-2. VH-Fc 1-16-3 DDC and VH-Fc E1-2 DDC worked efficiently in combination (with equivalent input) in CD22 positive tumor cells (Raji and Bjab) as well (**Figures 4E** **–G**). These data indicate that combination of PD-L1 therapy and CD22 therapy may achieve a superior effect to either alone. More extensive studies are required, but we present here an example of the potential applications of antibody VH domains as a therapeutic.

**Table 4:** SPR of CD22 specific VH domain E1-2.

Binders	KD (M)	Ka (1/Ms)	Ka Error	Kd (1/s)	Kd Error
E1-2	3.8 e-8	1.1 e5	3.5 e3	4.4 e-3	1.1 e-4

## Discussion

Construction of large size recombinant human domain libraries of high diversity and high aggregation resistance benefits target-specific binder selection, and addresses challenging barriers to finding effective therapeutics (9, 30). In the current study, we demonstrated our experience in construction of a large size VH domain library by optimizing the primers for CDR2/3 grafting into aggregation resistant scaffolds. CDR1 remained the same in scaffolds for several considerations: (1) CDR1 usually contributes less to antigen binding (31–33); (2) CDR2 and CDR3 grafting are sufficient for the target diversity of the library (26, 34, 35); (3) since the scaffolds selected are aggregation resistant, we intended to keep the original sequences unaltered as possible so as to minimize the risk of aggregation in subsequent binders isolated.

One common reason for antibody therapeutics failure in clinical trials is aggregation (36, 37), an essential bottleneck in the generation and production of antibody-based reagents for human therapeutics, especially smaller antibody formats that lack the inter-domain stabilization of their larger counterparts (25, 38, 39). We analyzed the binders we isolated by panning against TGFβ1 and MSLN, and to our experience, the output of binders from the improved library are superior to previous libraries we generated. Our results set an example for illustration of construction a large size VH domain library of high diversity and improved aggregation resistance.

1-16-3 is an aggregation resistant VH domain that targets human PD-L1. Different from the anti-PD-L1 antibodies currently available (40), the epitope of VH 1-16-3 is far away from the epitopes recognized by the natural PD1 ligand. This uncommon characteristic makes it possible for combination artorial applications with PD1/PD-L1 blockage drugs in the market. VH 1-16-3 showed a K_D_ of 48 nM by SPR, while VH-Fc 1-16-3 showed an EC_50_ less than 10 nM by ELISA. E1-2 is a CD22 specific VH domain with K_D_ of 38 nM and to our knowledge it is the first identified antibody VH domain specific for CD22. MMAE conjugated antibody domains are just one example of the application of domains as DDCs. These identified antibody VH domains of good biophysical properties that can be used for CAR-T constructs and other bi-specific antibody design, such as bispecific T cell engagers (BiTEs) (41, 42) and bispecific NK cell engagers (BiKEs) (43, 44).

Human antibody VH domains offer distinct advantages: (1) no need for further time-consuming humanization; (2) flexible administration such as lung delivery *via* inhalation for rapid deployment in response to SARS-CoV-2 (9, 45). Rapid progress has been made over the past few decades toward the development of therapeutic proteins (30). Human antibody VH domain is one of the fruition of such rapid progress of potent therapeutic proteins, and provides an exciting paradigm for the next generation of protein based therapeutics with their engineering potential and application advantages.

In summary, we demonstrated our strategy for construction of a large size human antibody VH domain library and briefly characterized the output binders from the current library. We characterized one PD-L1 specific VH domain 1-16-3 and one CD22 specific VH domain E 1-2 as DDCs *in vitro*. Our study provided key information for scientists who have interest in large size human domain library construction.

## Materials And Methods

### Human PBMCs

Fresh human normal peripheral blood mononuclear cells (PBMCs) (200 million cells/vial, CAT# SER-PBMC-200) were purchased from Zen-Bio, Inc. NC 27709. Consent and waiver was signed for purchasing purpose. Donor ages are ranging from 20 to 40 years old with unknown gender information. Fresh cells were directly used for mRNA extraction as previously described (4, 18).

### Expression and Purification of PD-L1 and CD22 Protein, VH Binders, and VH Bivalent Proteins

Full length human PD-L1 and CD22 sequences were synthesized by IDT (Coralville, Iowa) with sequence obtained from Uniprot (https://www.uniprot.org/uniprot/Q13421). The target sequences were cloned into an expression plasmid containing a CMV promotor, woodchuck posttranscriptional regulatory elements, and a His tag. Proteins were purified by Ni-NTA (GE Healthcare) chromatography. Target specific VH domains were cloned in pComb3x vector and purified from Escherichia coli HB2151 bacterial culture at 30°C in 2x YT medium (Y1003, Sigma-Aldrich) for 16 h with stimulation by 1 mM IPTG. Cells were lysed by Polymyxin B (Sigma-Aldrich). Lysate were spun down and supernatant was loaded over Ni-NTA (GE Healthcare). For conversion to Fc-fusion, the VH gene was re-amplified and re-cloned into pSectaq vector containing human Fc. VH-Fc proteins were expressed in the Expi293 expression system (Thermo Fisher Scientific) and purified with protein A resin (GenScript). Buffer replacement in protein purification used Column PD 10 desalting column (GE Healthcare). All protein purity was estimated as >95% by SDS-PAGE and protein concentration was measured spectrophotometrically (NanoVue, GE Healthcare). Further details can be found in our previous publication (4).

### Enzyme-Linked Immunosorbent Assays (ELISAs)

For ELISA assays, antigen protein was coated on a 96-well plate (Costar) at 50 ng/well in PBS overnight at 4°C. For the soluble VH binding assay, horseradish peroxidase (HRP)-conjugated mouse anti-FLAG tag antibody (A8592, Sigma-Aldrich) was used to detect VH binding. For detection of human Fc protein, HRP-goat anti-human IgG Fc secondary antibody (A18817, Thermo Fisher Scientific) was used. For the competition ELISA, 200 nM of VH-Fc 1-16 was incubated with serially diluted VH 1-16-3 proteins, and the mixtures were added to antigen-coated wells. After washing, competition was detected by HRP-goat anti-human IgG Fc secondary antibody (A18817, Thermo Fisher Scientific). All colors were developed by 3,3′,5,5′-tetramethylbenzidine (TMB, Sigma) and stopped by 1 M H_2_SO_4_ followed by recording absorbance at 450 nm by iMark™ Microplate Absorbance Reader (Bio-Rad Laboratories). Experiments were performed in duplicate and the error bars denote ± 1 SD.

### Surface Plasmon Resonance (SPR).

The kinetics of the antibody fragments were determined using a Biacore X100 (GE Healthcare). Human PD-L1/CD22 was (10 mg/mL) was immobilized onto a CM5 sensor chip (GE Healthcare, BR100012) by amine coupling. The antibody fragments diluted in HBS-EP buffer (10 mM HEPES, 150 mM NaCl, 3 mM EDTA, and 0.005% surfactant P20, pH 7.4) were injected over an immobilized surface (200 - 400 RU) for 180 sec at a rate of 20 µL/min, followed by dissociation for 600 sec. After each sample injection, the surface was regenerated by injection of regeneration solution (10 mM Glycine/10% Glycerol pH 2.0). The kinetic values, ka, kd, and KD were calculated using the BiacoreX100 Evaluation Software (GE Healthcare).

### Library Construction and Bio-Panning

A large phage-displayed human VH domain library was constructed by grafting of naturally occurring heavy-chain CDR2 s and CDR3 s (from PBMC pool of >30 healthy donors) to VH framework scaffolds. CDR2 s and CDR3 s were PCR amplified with the primers in **Table 2**. After library construction, 100 dandom clones were sequenced and no repeats were observe, indicating a good diversity of the library. The library was preabsorbed on streptavidin-M280-Dynabeads in phosphate-buffered saline (PBS) for 1 h at room temperature (RT) and incubated with 50 nM biotinylated PD-L1 or CD22 for 2 h at RT with gentle agitation. Phage particles binding to biotinylated antigen were separated from the phage library using streptavidin-M280-Dynabeads and a magnetic separator (Dynal). After washing 20 times with 1 ml of PBS containing 0.1% Tween-20 and another 20 times with 1 ml of PBS, bound phage particles were eluted by 100 mM triethanolamine followed by neutralization with 1 M, pH7.5 Tris-HCl. For the second round of panning, 10 nM (2 nM for the third round) of biotinylated protein was used as antigen. After the third round of panning, 96 individual clones were screened for binding to target protein by phage ELISA. Panels of VHs were selected, sequenced and characterized.

### Flow Cytometry

PD-L1 positive aAPC/CHO-K1 (Cat # J1205) cells and PD-L1 negative aAPC/CHO-K1 (Cat # J1191) were purchased from Promega. Bjab cells were purchased from (ABC-TC514S, AcceGen, New Jersey, USA). Raji cells (Cat# CCL-86) and Jurkat cells (TIB-152) were purchased from American Type Culture Collection (ATCC). Both T cells and B cells were maintained in ATCC-formulated RPMI-1640 Medium (ATCC 30-2001) with 10% fetal bovine serum (ATCC 30-2020). To confirm the cell surface binding of isolated antibody binders, cells were treated with VH 1-16, VH 1-16-3, E1-2, G10 or m971 for 1 h at 4°C and then stained with anti-DYKDDDDK Antibody, FITC (130-127-934, Miltenyi Biotec), or anti-DYKDDDDK (flag tag) Antibody, PE(130-101-577, Miltenyi Biotec), or PE anti-human IgG Fc Antibody (410707, BioLegend) for 0.5 h at 4°C. After each incubation with antibodies, cells were washed with cold PBS. Data were acquired using the flow cytometry BD LSR II (San Jose, CA) and analyzed by FlowJo 10.7.1.

### DDC Conjugations

Monomethyl auristatin E (MMAE) was conjugated to VH-Fc *via* the cross-linker OSu-Glu-VC-PAB (SET0100, Levena Biopharma, USA) with a molar ratio mAb : OSu-Glu-VC-PAB-MMAE of 1:10. The conjugation was performed in buffer composed of 50 mM potassium phosphate, 50 mM sodium chloride, and 2 mM EDTA (pH 6.5) with the reaction run for 18-24 h at room temperature. The reaction was stopped by adding 50 mM sodium succinate (pH 5.0) followed by buffer replacement by using Column PD 10 desalting column (GE Healthcare). The protein was washed and concentrated using 30 kDa Amicon centrifugal filter unit (Millipore Sigma CAT#UFC8030).

### Cell Viability Assays

Cell viability was measured using CellTiter-Glo or LDH-Glo (G7570, J2380, Promega). Briefly, CD22 positive or negative cells were plated into 96-wells, allowing attachment and growth for 24 hr, then triplicate wells were treated with DDCs, naked antibodies, free drugs, or DDCs plus competitor antibodies. Three to five days later, when untreated control wells were 70 to 90% confluent, reagent was added to the plates according to the supplier’s instructions. Wells treated identically but wells without cells were used to subtract background. Fluorescence (ex: 570 nm, Em: 585 nm) was measured using a CLARIOstar microplate reader (BMG Labtech) and data analyzed using GraphPad Prism 8.0.1 software. Significance was tested using one-way ANOVA, followed by the Tukey’s multiple *post hoc* test. ****P < 0.0001; ***P < 0.001; **P < 0.01; *P < 0.05 between the indicated groups. PD-L1 neutralization was done in a PD-1/PD-L1 Blockade Bioassay (J1250, Promega).

### Dynamic Light Scattering (DLS)

VH domains were buffer-changed to DPBS and filtered through a 0.22 μm filter. The concentration was adjusted to 5 mg/mL; 500 μL samples were incubated at 37°C. DLS measurement was performed on Zetasizer Nano ZS ZEN3600 (Malvern Instruments Limited, Westborough, MA) to determine the size distributions of protein particles.

### Epitope Mapping

Epitope mapping was performed by PEPperPRINT (Heidelberg, Germany). Briefly, a conformational PD-L1 peptide microarray was pre-stained with the secondary and control antibodies to investigate background interactions with the antigen-derived peptides that could interfere with the main assays. Subsequent incubation of conformational mesothelin peptide microarrays with human VH-Fc antibodies 1-16 at concentrations of 1 µg/ml, 10 µg/ml and 100 µg/ml in incubation buffer was followed by staining with the secondary and control antibodies. Read-out was performed with an Innopsys InnoScan 710-IR Microarray Scanner at scanning gains of 50/40 (red/green). The additional HA control peptides framing the peptide microarrays were simultaneously stained with the control antibody as internal quality control to confirm assay performance and peptide microarray integrity.

### Structural Modelling of PD-L1 VH Binder

PD-L1 structure was extracted from the PD1/PD-L1 complex (PDB ID:5IUS). VH1-structure was modeled by Swiss-model (46) followed by energy minimization. Z-DOCK program (https://zdock.umassmed.edu/) is used for docking VH1-16 onto PD-L1 (47). The antibody binding and blocking regions on PD-L1 were selected based on the experimental epitope mapping results. Z-DOCK outputed top 10 optimal poses, which are visually scrutinized for compatibility at the interaction interface and are avoiding of side chain clashes. We choose the most favorable pose as the binding model for further analysis. The structural figures are prepared by PyMol (48).

## Data Availability Statement

The original contributions presented in the study are included in the article/supplementary material. Further inquiries can be directed to the corresponding authors.

## Author Contributions

ZS designed the primers and constructed library, identified and characterized antibodies. ZS produced PD-L1 recombinant proteins and WL produced CD22 proteins and studied the modeling of epitope of antibody. ZS characterized DDCs. ZS wrote the first draft of the article. ZS and RO interpreted data. ZS, WL, JM, RO, and DD discussed the results and further revised the manuscript. All authors contributed to the article and approved the submitted version.

## Funding

This work was supported by the University of Pittsburgh Medical Center.

## Conflict of Interest

ZS, JM, and DD are co-inventors of a patent, filed on March 12 by the University of Pittsburgh, related to MSLN specific antibodies described in this paper. Binders against CD22 and PD-L1 are in invention disclosures in the University of Pittsburgh. Sequences of binders will be provided upon request and collaboration.

The remaining authors declare that the research was conducted in the absence of any commercial or financial relationships that could be construed as a potential conflict of interest.

## Publisher’s Note

All claims expressed in this article are solely those of the authors and do not necessarily represent those of their affiliated organizations, or those of the publisher, the editors and the reviewers. Any product that may be evaluated in this article, or claim that may be made by its manufacturer, is not guaranteed or endorsed by the publisher.
